# Supplementary material for: Prevalence and associated factors of mental health disorders among Brazilian healthcare workers in times of the COVID-19 pandemic: A web-based cross-sectional study
Source: PLoS One. 2023 Jun 6;18(6):e0274927. doi: 10.1371/journal.pone.0274927 (PMC10243644; doi:10.1371/journal.pone.0274927)
Supplement: S2 File — (DOCX) [file pone.0274927.s002.docx]

Ethics Statement

Informed consent was obtained electronically from all participants, with the approval of the Fiocruz Brasília Ethics Committee (n. 4.401.333).
