# Supplementary material for: Prevalence and associated factors of mental health disorders among Brazilian healthcare workers in times of the COVID-19 pandemic: A web-based cross-sectional study
Source: PLoS One. 2023 Jun 6;18(6):e0274927. doi: 10.1371/journal.pone.0274927 (PMC10243644; doi:10.1371/journal.pone.0274927)
Supplement: S3 File — (PDF) [file pone.0274927.s003.pdf]

Fiocruz Mato Grosso do Sul
Education Area

September 2, 2022.
To: Emily Chenette
Editor-in-Chief

Subject: Fund request for publication

Our research group has submitted the manuscript "**Self-perception of mental health among Brazilian healthcare workers: a web-based cross-sectional study.**" to be considered for publication at *PLOS ONE* .The article authors are: Silvia Helena Mendonça de Moraes, Inara Pereira da Cunha, Everton Ferreira Lemos, Maria de Lourdes Oshiro, Lesly Lidiane Ledezma Abastoflor, Rosana Teresinha D`Orio de Athayde Bohrer, Vicente Sarubbi Jr, Fabrícia Barros de Souza, Débora Dupas Gonçalves do Nascimento, Sandra Maria do Valle Leone de Oliveira.

PLOS ONE is recognized as one of the most important scientific journals in the world and ideal for the dissemination of the results of our research. We know there are costs for the editorial working of the journals. However, at the moment, Brazil faces a serious financial crisis with deep cuts in the financing research and publication of the results. Due to an economic downturn generated by COVID-19 in Brazil, the $1,805 fee required for the publication is equivalent to one-month salary of a researcher in Brazil.

Because of Brazil's mounting economic woes, federal funding for science and technology is now at its lowest level in modern history, dropping by more than half over the past 5 years.

The veracity of this information can be verified by many reports in the international media such as:

https://www.nature.com/articles/d41586-019-02484-w
https://www.scidev.net/global/news/scientists-protest-budget-cuts-in-brazil/
https://namidia.fapesp.br/scientists-protest-budget-cuts-in-brazil/244877
https://www.scielo.br/scielo.php?pid=S0001-37652020000700202&script=sci_arttext
http://www.alternautas.net/blog/2020/9/9/culture-war-against-brazilian-universities-how-budget-cuts-and-changes-in-tertiary-education-policies-are-affecting-the-academic-community
https://www.sciencemag.org/news/2021/04/hostile-environment-brazilian-scientists-face-rising-attacks-bolsonaro-s-regime
https://www.theguardian.com/education/2021/mar/19/this-happens-in-brazil-not-britain-academics-in-despair-as-global-research-funds-pulled

We believe that lack of funds should not be a barrier to the publication of open access. In our case, the cost of the publication would be paid by the author's researcher salaries. A discount on the publication fee would encourage us to continue being researchers even in the face of financial difficulties.

In addition, the Institutions of all authors at the moment have a minimal institutional fee support fund to research, just to pay photocopies for questionnaires or some consumption products for laboratories, not to pay for publications.

Yours Sincerely,

Jislaine de Fátima Guilhermino
SIAPE: 1355012
FIOCRUZ - VPGDI

Reseacher Jislaine de Fátima Guilhermino
Director of Fiocruz Mato Grosso do Sul
